# Late subsequent leukemia after childhood cancer: A report from the Childhood Cancer Survivor Study (CCSS)

**DOI:** 10.1002/cam4.70086

**Published:** 2024-10-21

**Authors:** Taumoha Ghosh, Geehong Hyun, Rikeenkumar Dhaduk, Miriam Conces, Michael A. Arnold, Rebecca M. Howell, Tara O. Henderson, Aaron McDonald, Leslie L. Robison, Yutaka Yasui, Kirsten K. Ness, Gregory T. Armstrong, Joseph P. Neglia, Lucie M. Turcotte

**Affiliations:** ^1^ Primary Children's Hospital/University of Utah Salt Lake City Utah USA; ^2^ St. Jude Children's Research Hospital Memphis Tennessee USA; ^3^ Nationwide Children's Hospital Columbus Ohio USA; ^4^ Children's Hospital of Colorado Aurora Colorado USA; ^5^ University of Colorado, Anschutz Medical Campus Aurora Colorado USA; ^6^ The University of Texas MD Anderson Cancer Center Houston Texas USA; ^7^ University of Chicago Chicago Illinois USA; ^8^ University of Minnesota Minneapolis Minnesota USA

**Keywords:** childhood cancer, epipodophyllotoxins, late effect, subsequent leukemia

## Abstract

**Background:**

Subsequent short‐latency leukemias are well‐described among survivors of childhood cancer. However, late (5–14.9 years from diagnosis, LL) and very late (≥15 years from diagnosis, VLL) subsequent leukemias have not been well studied. We assessed risk factors, prevalence, and outcomes for LL and VLL in the Childhood Cancer Survivor Study cohort.

**Methods:**

Subsequent leukemias, among 25,656 five‐year survivors, were self‐reported and confirmed by pathology review. Standardized incidence ratios (SIR) and cumulative incidences were calculated, and relative risks (RR) were estimated using Cox regression for exposures.

**Results:**

Seventy‐seven survivors developed subsequent leukemia, 49 survivors with LL (median time from diagnosis 7.8 years, range 5.0–14.5 years) and 28 with VLL (median time from diagnosis 25.4 years, range 15.9–42.8 years), with a cumulative incidence of 0.23% (95% CI 0.18%–0.30%) 20 years from diagnosis for all subsequent leukemias. The most common leukemia subtypes were acute myeloid leukemia, myelodysplastic syndrome, and chronic myeloid leukemia. Compared to the general population, survivors were at increased risk, for developing LL (SIR 9.3, 95% CI 7.0–12.1) and VLL (SIR 5.9, 95% CI 3.9–8.4). In multivariable relative risk analyses, cumulative epipodophyllotoxin dose >4000 mg/m^2^ was associated with increased risk for LL and VLL (RR 4.5, 95% CI 2.0–9.9).

**Conclusions:**

In this large series of late subsequent leukemias, survivors of childhood cancer are at increased risk, with no evidence of plateau over time. We observed most risk among survivors who received high cumulative doses of epipodophyllotoxins. Ongoing consideration for this late effect should continue beyond 10 years.

## Graphical abstract

Although subsequent short‐latency leukemias are well described‐among survivors of childhood cancer, late (5–14.9 years from diagnosis) and very late (>15 years from diagnosis) have not been well studied. We assessed prevalence, risk factors, and outcomes for late and very late subsequent leukemias in the Childhood Cancer Survivor Study (CCSS) cohort. In this large number of observed late and very late leukemias among survivors of childhood cancer, we observed that the risk for subsequent leukemia persisted beyond 10 years, which was a previously established plateau in the literature. We hope that this study will stimulate ongoing research into the need for additional screening recommendations for survivors of childhood cancer as they continue to age.

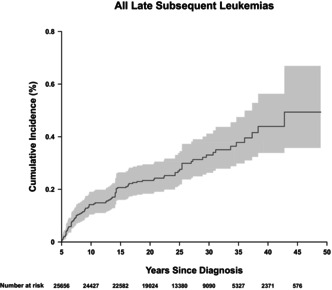

## 1 INTRODUCTION

Five‐year survival following a childhood cancer diagnosis exceeds 85%, and approximately half a million pediatric cancer survivors are now alive in the United States.^1^, ^2^ As such, the identification of late effects after cancer treatment, including subsequent malignant neoplasms (SMN)^3^ and chronic medical conditions,^4^ is critical to providing appropriate and necessary care to long‐term survivors.^5^ After primary cancer recurrence, SMNs account for the highest proportion of deaths in the survivor population.^6^, ^7^

Subsequent leukemias, both of the lymphoid and myeloid lineages, have been well‐described in survivors of pediatric cancer and have among the shortest latency of SMNs in long‐term survivors, commonly also referred to as therapy‐ or treatment‐related leukemias.^8^, ^9^, ^10^ They are most frequently associated with exposures to certain classes of chemotherapies, including topoisomerase II inhibitors and alkylating agents. Topoisomerase II inhibitor exposure is most frequently associated with early‐occurring (median 2–3 years from exposure) subsequent leukemias that characteristically harbor *MLL* gene rearrangements,^11^ whereas alkylating agent associated leukemias are observed later (median 4–7 years from exposure) and are most typically associated with genetic alterations involving chromosomes 5 and 7.^12^, ^13^, ^14^ Importantly, among large cohorts of survivors of childhood cancer, as the length of follow up increases, there is growing recognition that late‐occurring subsequent leukemia is more common than previously appreciated. This was previously described within the Childhood Cancer Survivor Study (CCSS), where an overall 6.3‐fold increased risk was reported for subsequent leukemia among survivors and the risk remained 3.5‐fold increased at ≥15 years after their primary childhood cancer diagnoses.^15^, ^16^ The PanCareSurFup cohort examined risk for subsequent primary leukemias and found an overall 3.7‐fold increased risk for subsequent leukemia among survivors that remained elevated (SIR 2.4) past 20 years from primary childhood cancer diagnosis.^17^ Although both analyses demonstrated long‐term risk for subsequent leukemia among survivors of childhood cancer, neither presented treatment‐associated risk factors due to limitations in the number of cases^15^ and availability of comprehensive treatment data.^17^

Following the prior subsequent leukemia report, the CCSS expanded its cohort and now includes individuals diagnosed and treated for childhood cancer between 1970 and 1999. Additionally, there have been 9 years of follow‐up from the time of the previous publication. This study presents an important opportunity to expand understanding of treatment‐associated risk factors for late (5–14.9 years from diagnosis) and very late (≥15 years from diagnosis) subsequent leukemia.

## 2 MATERIALS/METHODS

The CCSS is a retrospective cohort study with long‐term follow‐up of survivors of childhood cancer who are past five‐years from initial diagnosis, and diagnosed between 1970 and 1999 at one of 31 participating institutions in the United States and Canada. Participants were eligible to enter the cohort if younger than 21 years of age when diagnosed with initial cancer (leukemia, Hodgkin lymphoma [HL], non‐Hodgkin lymphoma [NHL], central nervous system cancer, renal cancer, neuroblastoma, rhabdomyosarcoma, or bone cancer). All protocol and contact documents were accepted and validated by human subjects' committees at participating institutions. Informed consent was obtained from participants or their parents if they were minors. Minor participants were reconsented at age of majority. The CCSS methodology has been formerly reported upon.^18^, ^19^

Subsequent leukemia cases occurring five or more years after childhood cancer diagnosis were discovered by self‐report and verified by pathology report review, or if unavailable, medical record and/or death certificate review. Cases of subsequent leukemia were classified as either late leukemia (LL; ≥5–14.9 years from diagnosis) or very late leukemia (VLL; ≥15 years from diagnosis), as had been established by Nottage et al.^15^ due to the previous understanding that late leukemia risk plateaued between 10 and 15 years from initial diagnosis. Neoplasms considered eligible for inclusion including International Classification of Diseases of Oncology (ICD‐O‐3) codes were leukemia, not otherwise specified (NOS) (9800/3), acute undifferentiated leukemia (9801/3), acute biphenotypic leukemia (9805/3), B‐lymphoblastic leukemia/lymphoma (NOS) (9811/3, 9835/3, 9836/3), lymphoid leukemia NOS (9820/3), chronic lymphocytic leukemia/small lymphocytic lymphoma (9823/3), Burkitt cell leukemia (9826/3), T‐cell leukemia/lymphoma (9827/3, 9831/3, 9837/3), acute myeloid leukemia (AML) (9861/3, 9866/3, 9867/3, 9871/3, 9872/3, 9874/3, 9891/3), myelodysplastic syndrome (9980/3, 9983/3, 9985/3), and chronic myeloid leukemia (CML), NOS (9863/3). Relapse of primary leukemia, based on comparison of pathology reports by the pathologist and oncologist team that reviews all CCSS subsequent neoplasm reports and again by the study authors, were considered recurrences rather than subsequent leukemia, and were excluded (*n* = 356). Leukemias that occurred >20 years from the childhood leukemia diagnosis, were assumed to be a secondary neoplasm (*n* = 1), per previously established standard procedures within the CCSS SMN review process. Demographic characteristics and cancer therapies received following childhood cancer diagnosis, including surgery (splenectomy), hematopoietic cell transplantation (HCT), chemotherapy, and radiation, were ascertained through abstraction of medical records.^19^, ^20^ Specifically, chemotherapy exposures, including cumulative doses of anthracyclines (doxorubicin equivalents),^21^, ^22^ platinating agents,^23^, ^24^ and alkylating agents (cyclophosphamide equivalent doses)^25^ were abstracted. Radiation exposure was evaluated by body site (cranial irradiation, total body irradiation, other body site irradiation) and maximum dose received, which was taken as the summation of the delivered doses from all overlapping fields in each body region.^26^ Cytogenetic data, where available, were ascertained via medical record abstraction. Smoking status was also determined from self‐report within baseline and follow‐up questionnaires of participants who were adults at the time of questionnaires.^27^ If they had ever smoked >100 cigarettes in their lifetime, participants were considered smokers. All others were presumed to have “never” smoked.

Descriptive statistical analysis for the comparison of three groups, late, very late leukemia, and without subsequent leukemia, was performed regarding the distributions of demographic and clinical characteristics at diagnosis, treatment characteristics, smoking status, and vital status by using chi‐square or one‐way analysis of variance tests. The cumulative incidence of subsequent leukemias was estimated from 5 years after diagnosis to the first occurrence of subsequent leukemia, death was treated as a competing risk and at the date of last contact was censored, stratifying by the primary cancer diagnosis and treatment exposures (epipodophyllotoxin, alkylating agent and radiation). To compare the rate of subsequent leukemia events in the CCSS cohort with the rates in the U.S. population, standardized incidence ratios (SIRs), that is the ratio of the observed to expected number of events, and absolute excess risks (AERs), which is calculated by deducting the expected number of events from the observed number of events divided by person‐years at risk and multiplied by 1000, were calculated with data from the Surveillance, Epidemiology and End Results (SEER) database using age‐, sex‐, race, and calendar‐year‐specific incidence rates of leukemia.^2^ Leukemia incidence data of the SEER population, Incidence—SEER Research Data, 9 Registries, Nov 2020 Sub (1975–2018), was downloaded using SEER*Stat, including the number of malignant cases and the population size in each of the strata defined jointly by age, sex, race, and calendar year.

Multivariable Cox regression models, with time to subsequent leukemia event since primary cancer diagnosis as the time scale, were used to evaluate for associations between survivor characteristics and the risk of subsequent leukemia, where the analyses were limited to survivor characteristics and treatment variables with univariate association at p‐value less than or equal to 0.2. All‐cause mortality risks were estimated using multivariable Cox regression models, adjusted for sex and attained age. The proportional hazards assumptions were assessed with the Kolmogorov‐type supremum test and the assumptions were not violated. Sampling weights were applied in all analyses to account for under‐sampling of acute lymphoblastic leukemia (ALL) survivors diagnosed 1987–1999. All statistical tests were two‐sided and *p*‐values less than 0.05 were determined to be statistically significant. SAS (version 9.4) was used for statistical analysis and R (version 4.2.2) was used for figures.

## 3 RESULTS

Within the group of the 25,656 survivors included in the study, 77 were diagnosed with a subsequent leukemia. Of those, 49 survivors were diagnosed with late leukemia (LL) and 28 survivors were diagnosed with very late leukemia (VLL). Demographic and treatment factors of survivors who developed subsequent LL versus subsequent VLL versus those who did not develop subsequent leukemia (NL) were assessed (Table 1). Mean age at primary diagnosis differed among the three groups, with those who developed very late leukemia being older at primary diagnosis (median 13 years (VLL) vs. 8 years (LL) vs. 6 years (NL), *p =* 0.001). No difference was seen in the distribution of sex or race/ethnicity. Among survivors who developed LL, the most common primary diagnoses were ALL, HL, and NHL and among those who developed VLL, HL, ALL, and osteosarcoma. There were differences in epidophyllotoxin (25.0% [LL] vs. 12.6% [VLL] vs. 5.6% [NL], *p* < 0.001) and alkylating agent (44.5% [LL] vs. 29.5% [VLL] vs. 23.4% [NL], *p =* 0.018) exposures between groups. Those who developed LL received cranial or total body irradiation more frequently than the other two groups (48.5% [LL] vs. 28.9% [VLL] vs. 29.1% [NL], *p =* 0.005), and were more likely to have undergone HCT (30.3% [LL] vs. 0% [VLL] vs. 5.7% [NL], *p* < 0.001). Those who developed VLL were more likely to have ever smoked than the other two groups (29.1% [VLL] vs. 3.4% [LL] vs. 11.8% [NL], *p* = 0.001), though the absolute numbers of those who smoked were quite low, making further analyses of smoking status' impact on the development of subsequent leukemia not feasible. Survivors who experienced subsequent leukemia were more likely to be deceased compared to those who did not (71.2% [LL] vs. 49.3% [VLL] vs. 14.4% [NL], *p* < 0.001).

**TABLE 1:** Characteristics of survivors with and without late leukemia.^a^

	Survivors with late (≥5–14.9 years from dx) leukemia *N* = 49		Survivors with very late (≥15 years from dx) leukemia *N* = 28		Survivors without late leukemia *N*		
	Median	Range	Median	Range	Median	Range	*p*‐Value
Mean age at primary diagnosis, years	8.00	(0, 20)	13.00	(0, 20)	6.00	(0, 20)	0.001
	***N***	**%**	***N***	**%**	***N***	**%**	
Age at primary diagnosis, years							
0–4 years	13	34.85	5	24.76	10,169	42.69	0.002
5–9 years	11	22.50	3	9.73	5770	23.74	
10–14 years	13	22.50	8	26.62	5420	19.15	
≥15 years	12	20.15	12	37.90	4220	14.43	
Sex							
Male	28	56.30	19	70.14	13,674	53.53	0.165
Female	21	43.70	9	29.86	11,905	46.47	
Race and ethnicity							
White	39	78.80	21	76.62	20,419	79.07	0.328
Black	4	10.95	2	6.48	1623	6.43	
Hispanic	3	5.30	5	16.89	2037	8.78	
Other/Unknown	3	4.95	0	0	1500	5.73	
Decade of diagnosis							
1970–79	11	18.15	11	35.66	6590	22.28	0.035
1980–89	20	37.35	13	50.69	10,012	36.82	
1990–99	18	44.49	4	13.65	8977	40.90	
Childhood cancer diagnosis							
ALL	14	42.24	5	25.44	6596	35.83	0.023
AML	1	1.65	2	6.48	924	3.12	
Other leukemia	1	1.65	1	3.24	328	1.11	
Astrocytomas	2	3.30	1	3.24	2685	9.08	
Medulloblastoma	2	3.30	0	0	1038	3.51	
Other CNS	0	0	0	0	761	2.57	
Hodgkin lymphoma	13	21.45	9	29.18	3085	10.43	
NonHodgkin lymphoma	6	9.90	3	9.73	2105	7.12	
Kidney	0	0	0	0	2274	7.69	
Neuroblastoma	5	8.25	1	3.24	1940	6.56	
Soft tissue sarcoma	1	1.65	2	6.48	1753	5.93	
Ewing sarcoma	1	1.65	1	3.24	736	2.49	
Osteosarcoma	3	4.95	3	9.73	1251	4.23	
Other bone tumor	0	0	0	0	103	0.35	
Chemotherapy							
Anthracycline (mg/m^2^)							
None	18	36.33	12	43.85	12,055	48.51	0.220
1–100	6	28.50	1	13.06	2180	15.51	
101–300	10	20.61	8	28.73	5482	24.54	
>300	7	14.55	4	14.36	2845	11.43	
Epipodophyllotoxin (mg/m^2^)							
None	28	55.59	24	83.93	19,035	80.09	<0.001
1–1000	3	6.33	0	0	1092	4.96	
1001–4000	4	13.08	1	3.47	1949	9.31	
>4000	7	25.01	1	12.61	895	5.63	
Alkylating agent (CED) (mg/m^2^)							
None	10	27.75	13	51.11	10,662	48.13	0.018
1–3999	5	10.98	3	11.61	2840	16.10	
4000–7999	7	16.76	2	7.74	2822	12.41	
8000+	15	44.52	5	29.54	5395	23.36	
Platinum agents (mg/m^2^)							
None	38	87.03	26	100.0	20,679	90.86	0.221
1–400	3	5.56	0	0	995	3.68	
401–750	1	1.85	0	0	993	3.67	
>750	3	5.56	0	0	486	1.79	
Radiation							0.005
None	12	28.83	14	47.61	10,398	48.75	
Cranial radiation	15	38.68	6	28.94	6535	26.47	
Total body irradiation	5	9.84	0	0	600	2.67	
Other radiation site	12	22.66	7	23.45	5948	22.12	
Maximum radiation dose to anybody region (Gy) (range)	23.00	(0, 55.00)	15.00	(0, 66.00)	0	(0, 309.00)	0.101
Splenectomy							
Yes	7	11.55	6	19.45	1471	4.99	<0.001
No	42	88.45	22	80.55	24,108	95.01	
Hematopoietic cell transplantation							
None	33	69.70	28	100.0	24,020	94.32	<0.001
Autologous	9	20.23	0	0	1169	4.75	
Allogeneic	6	10.07	0	0	259	0.93	
Smoking status							
Nonsmoker	26	96.65	20	67.48	17,568	74.22	0.001
Current smoker	0	0	1	3.37	3304	13.93	
Ever smoked	1	3.35	6	29.14	2750	11.84	
Vital status							
Alive	12	28.85	13	50.69	21,486	85.61	<0.001
Deceased	37	71.15	15	49.31	4093	14.39	
Survival after childhood cancer diagnosis, years							
5–9	24	49.35	0	0	1079	3.99	<0.001
10–14	10	16.50	0	0	1686	6.61	
15–19	4	6.95	4	13.65	3245	13.29	
20–24	4	15.65	4	12.97	5712	24.45	
25–29	2	3.30	4	12.97	4404	18.55	
30–34	2	3.30	7	31.24	3780	13.94	
> = 35	3	4.95	9	29.18	5673	19.18	
	**Median**	**Range**	**Median**	**Range**	**Median**	**Range**	
Number of person‐years since cohort entry	7.33	(0.41, 31.85)	26.30	(12.01, 40.67)	19.90	(0.008, 43.94)	<0.001
Mean years of follow up from diagnosis, years	14.01	(5.41, 36.85)	30.43	(17.00, 45.67)	25.82	(5.00, 48.93)	<0.001

^a^ Since acute lymphoblastic leukemia survivors diagnosed 1987–1999 were under‐sampled in the CCSS cohort, sampling weights were applied for all percentages and means/medians, using a weight of 1.21 for those aged 0 or 11–20 years at diagnosis and a weight of 3.63 for those aged 1–10 years.

Characteristics of subsequent leukemia cases are described in Table 2. Median time to diagnosis of LL and VLL from time of initial diagnosis was 7.8 years (range 5.0–14.5) and 25.4 years (range 15.9–42.8), respectively. The most common histologic LL diagnoses were AML, myelodysplastic syndrome (MDS) and CML. The most common VLL diagnoses were AML, MDS, and ALL. The distribution of histologic diagnoses of LL compared to VLL was similar (*p =* 0.504). Cytogenetic data was available for 50.6% (*n* = 39) of subsequent leukemia cases. Among those with available cytogenetic data, three had normal cytogenetics, 15 had complex cytogenetics, and 21 had single cytogenetic abnormalities, including 15 with translocations. Interestingly nine of the translocations were t (9;22) in the setting of secondary CML, and only three involved 11q23. Among survivors who developed subsequent leukemia, only three had an underlying genetic predisposition syndrome (Trisomy 21, *n* = 1; Nevoid Basal Cell Carcinoma Syndrome, *n* = 1; “Other” genetic disorder, *n* = 1). Finally, among survivors who developed subsequent leukemia, seven had a prior history of SMN, for a total of nine SMN events (breast cancer, *n* = 5; bladder papillary transitional cell carcinoma, *n* = 1; endometroid adenocarcinoma, *n* = 1; osteosarcoma, *n* = 2).

**TABLE 2:** Histologic type of leukemia, and survival characteristics of subsequent leukemia cases.

	Survivors with late (≥5–14.9 years from dx) leukemia diagnosis *N* = 49	Survivors with very late (≥15 years from dx) leukemia diagnosis *N* = 28	*p*‐Value
Time from childhood cancer diagnosis to leukemia diagnosis, years Median time (range)	7.78 (5.04, 14.49)	25.42 (15.92, 42.79)	—
5–10	36 (69.50)	0 (0.0)	
10.1–15	13 (30.50)	0 (0.0)	
15.1–20	0 (0.0)	7 (23.38)	
>20	0 (0.0)	21 (76.62)	
Age at leukemia diagnosis, years Median age (range)	18.40 (5.70, 32.19)	36.37 (18.21, 58.05)	—
5–10	9 (23.90)	0 (0.0)	
11–20	22 (45.35)	1 (3.24)	
21–30	17 (29.10)	7 (31.92)	
31–40	1 (1.65)	8 (25.93)	
41–50	0 (0.0)	8 (25.93)	
51–60	0 (0.0)	4 (12.97)	
Leukemia diagnosis			
Acute leukemia NOS	2 (3.3)	2 (6.48)	0.504
Acute lymphoblastic leukemia	5 (8.25)	5 (16.21)	
Acute myeloid leukemia	25 (51)	14 (54.61)	
Chronic myeloid leukemia	6 (18.6)	3 (9.73)	
Myelodysplastic syndrome	11 (18.85)	4 (12.97)	
Survival after leukemia diagnosis, years			
0–5	35 (67.85)	21 (77.31)	0.612
6–10	5 (16.95)	3 (9.73)	
11–15	2 (3.65)	2 (6.48)	
16–26	7 (11.55)	2 (6.48)	
Cause of death			
Leukemia	20 (60.08)	9 (60.55)	0.909
Ardiac	1 (2.32)	0 (0.0)	
Other SMN	7 (16.24)	5 (32.87)	
Recurrent	2 (4.64)	0 (0.0)	
External	1 (2.81)	0 (0.0)	
Other causes	2 (4.64)	0 (0.0)	
Unknown	4 (9.28)	1 (6.57)	

**FIGURE 1:**
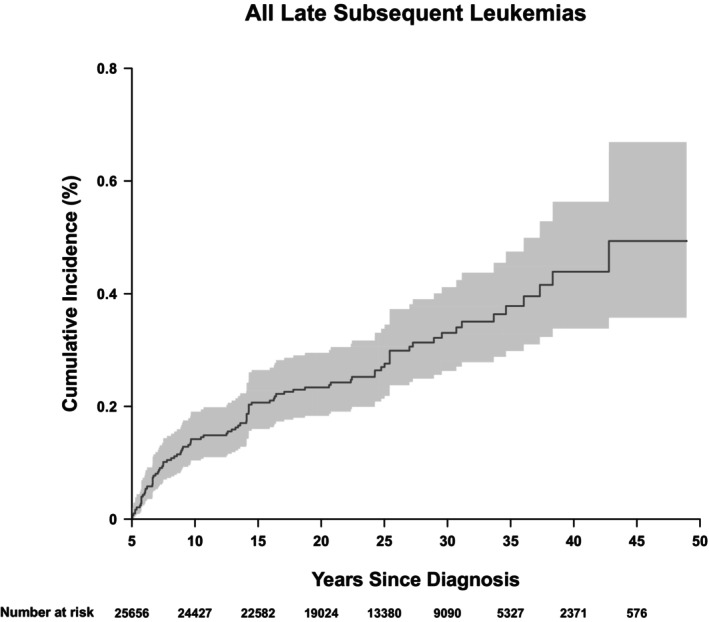
Cumulative Incidence Plot of Subsequent Leukemia. The 20‐year cumulative incidence of subsequent leukemia was 0.23% (95% CI 0.18%–0.30%) and does not show evidence of plateau.

The 20‐year cumulative incidence of subsequent leukemia was 0.23% (95% CI 0.18%–0.30%) and does not show evidence of plateau (Figure 1). A nine‐fold greater risk for developing subsequent late leukemia was noted in survivors within the cohort compared to the general population (SIR 9.3, 95% CI 7.0–12.1). The absolute excess risk (AER) was 0.09 per 1000 person‐years (95% CI 0.07–0.12) (Table 3). For VLL, survivors had a near six‐fold greater risk for developing subsequent very late leukemia compared to the general population (SIR 5.9, 95% CI 3.9–8.4) and the absolute excess risk (AER) was 0.04 per 1000 person‐years (95% CI 0.03–0.07) (Table 3). SIRs for LL were highest among survivors of ALL (SIR 14.2, 95% CI 9.2–20.8), medulloblastoma (SIR 12.7, 95% CI 1.4–45.7), HL (SIR 10.2, 95% CI 4.9–18.8), and NHL (SIR 14.0, 95% CI 5.1–30.4), whereas SIRs for VLL were highest among survivors of AML (SIR 11.6, 95% CI 1.3–41.8), osteosarcoma (SIR 10.9, 95% CI 2.2–31.9), and HL (SIR 8.8, 95% CI 3.8–17.3).

**TABLE 3:** Cumulative incidence at 20 years, SIR, and AER per 1000 person years for subsequent leukemia (by childhood cancer diagnosis and/or treatment exposures)^a^ ^,^ ^b^.

Characteristic	Survivors with late (≥5–14.9 years from dx) leukemia diagnosis *N* = 49					Survivors with very late (≥15 years from dx) leukemia diagnosis *N* = 28				
	Number observed	Number expected	SIR (95% CI)	AER (95% CI)	Cumulative Incidence % (95% CI)	Number observed	Number expected	SIR (95% CI)	AER (95% CI)	Cumulative Incidence % (95% CI)
All cases	55.6	6.0	9.3 (7.0, 12.1)	0.09 (0.07, 0.12)	0.20 (0.16, 0.26)	28.8	4.9	5.9 (3.9, 8.4)	0.04 (0.03, 0.07)	0.03 (0.01, 0.05)
ALL	25.6	1.8	14.2 (9.2, 20.8)	0.12 (0.08, 0.18)	0.25 (0.16, 0.36)	6.8	1.4	4.9 (1.9, 10.1)	0.03 (0.01, 0.06)	0.02 (0.01, 0.08)
AML	1	0.2	4.8 (0.1, 26.9)	0.05 (0, 0.15)	0.11 (0.01, 0.59)	2	0.2	11.6 (1.3, 41.8)	0.10 (0, 0.26)	0 (NA)
Other leukemia	1	0.1	11.9 (0.2, 66.2)	0.18 (0, 0.93)	0.32 (0.03, 1.67)	1	0.1	13.4 (0.2, 74.8)	0.18 (0, 0.93)	0 (NA)
Astrocytomas	2	0.5	4.1 (0.5, 14.9)	0.03 (0, 0.14)	0.07 (0.02, 0.26)	1	0.4	2.5 (0, 13.8)	0.01 (0, 0.10)	0 (NA)
Medulloblastoma	2	0.2	12.7 (1.4, 45.7)	0.10 (0.01, 0.36)	0.19 (0.04, 0.67)	0	0.1	—	—	0 (NA)
Other CNS	0	0.1	—	—	0 (NA)	0	0.1	—	—	0 (NA)
Hodgkin lymphoma	10	1.0	10.2 (4.9, 18.8)	0.16 (0.07, 0.31)	0.42 (0.24, 0.71)	8	0.9	8.8 (3.8, 17.3)	0.13 (0.05, 0.26)	0.10 (0.03, 0.29)
NonHodgkin lymphoma	6	0.4	14.0 (5.1, 30.4)	0.14 (0.05, 0.31)	0.29 (0.12, 0.60)	3	0.4	8.0 (1.6, 23.5)	0.07 (0.01, 0.20)	0 (NA)
Kidney	0	0.4	—	—	0 (NA)	0	0.3	—	—	0 (NA)
Neuroblastoma	4	0.4	10.7 (2.9, 27.4)	0.10 (0.02, 0.27)	0.26 (0.10, 0.58)	1	0.3	3.7 (0, 20.4)	0.02 (0, 0.13)	0.06 (0.01, 0.33)
Soft tissue sarcoma	0	0.4	—	—	0.06 (0, 0.32)	2	0.3	6.3 (0.7, 22.9)	0.05 (0, 0.20)	0 (NA)
Ewing sarcoma	1	0.2	6.2 (0.1, 34.3)	0.06 (0, 0.36)	0.14 (0.01, 0.74)	1	0.1	6.9 (0.1, 38.3)	0.07 (0, 0.36)	0.15 (0.01, 0.79)
Osteosarcoma	3	0.3	10.0 (2.0, 29.2)	0.11 (0.02, 0.34)	0.24 (0.07, 0.68)	3	0.3	10.9 (2.2, 31.9)	0.11 (0.02, 0.34)	0 (NA)
Other bone tumor	0	0.04	—	—	0 (NA)	0	0.04	—	—	0 (NA)
Treatment exposures										
Epipodophyllotoxin										
Yes	25.8	1.0	25.2 (16.4, 37.0)	0.25 (0.16, 0.36)	0.45 (0.30, 0.65)	4.6	0.8	5.6 (1.7, 13.4)	0.04 (0.01, 0.09)	0.02 (0.00, 0.11)
No	26.2	4.5	5.8 (3.8, 8.5)	0.05 (0.03, 0.08)	0.13 (0.09, 0.19)	22.2	3.7	5.9 (3.7, 9.0)	0.05 (0.03, 0.07)	0.03 (0.01, 0.06)
Alkylating agent										
Yes	39.3	3.0	13.1 (9.3, 17.9)	0.13 (0.09, 0.18)	0.28 (0.20, 0.38)	14.6	2.5	5.8 (3.2, 9.6)	0.05 (0.02, 0.08)	0.02 (0.01, 0.06)
No	12.6	2.5	5.0 (2.7, 8.7)	0.04 (0.02, 0.08)	0.10 (0.06, 0.18)	12.2	2.0	6.0 (3.1, 10.4)	0.04 (0.02, 0.08)	0.03 (0.01, 0.08)
Radiation										
Yes	35.7	3.0	11.8 (8.3, 16.4)	0.13 (0.09, 0.18)	0.27 (0.20, 0.37)	14.6	2.5	5.8 (3.2, 9.6)	0.05 (0.02, 0.08)	0.02 (0.01, 0.07)
No	15.3	2.5	6.0 (3.4, 9.9)	0.05 (0.03, 0.09)	0.11 (0.07, 0.19)	13.2	2.1	6.4 (3.4, 10.9)	0.05 (0.02, 0.08)	0.03 (0.01, 0.08)

^a^ Since acute lymphoblastic leukemia survivors diagnosed 1987–1999 were under‐sampled, sampling weights, where a weight of 1.21 for those aged 0 or 11–20 years at diagnosis and a weight of 3.63 for those aged 1–10 years, were applied.

^b^ SEER data population of “Incidence—SEER Research Data, 9 Registries, Nov 2020 Sub (1975‐2018)” was used to calculate SIRs.

Multivariate analyses to assess relative rates of subsequent leukemia by demographic and treatment characteristics were performed and included characteristics, which had a *p*‐value ≤ 0.2 on univariate analyses (Table 4). Older age (≥15 years) at initial diagnosis (relative risk [RR] 2.21, 95% CI 1.08–4.51), high cumulative epipodophyllotoxin dose (>4000 mg/m^2^; RR 4.51, 95% CI 2.04–9.94), and treatment with HCT (RR 5.37, 95% CI 2.72–10.62) were associated with increased risk for subsequent leukemia. When LL and VLL were considered separately (Table 4), high cumulative epipodophyllotoxin dose (RR 4.73, 95% CI 1.76–12.72) and treatment with HCT (RR 8.48, 95% CI 3.98–18.05) were associated with LL risk, and older age at diagnosis (RR 4.44, 95% CI 1.45–13.59), and high cumulative dose epipodophyllotoxin exposure (RR 5.08, 95% CI 1.27–20.37) were risk factors for VLL.

**TABLE 4:** Multivariable analyses of risk factors for subsequent leukemia.

Characteristic	Late (≥5–14.9 years from dx) leukemia			Very late (≥15 years from dx) leukemia			All subsequent leukemias		
	Relative Risk (95% CI)		*p*‐Value	Relative Risk (95% CI)		*p*‐Value	Relative Risk (95% CI)		*p*‐Value
Gender									
Male	1.32	(0.71, 2.45)	0.374	2.10	(0.92, 4.81)	0.078	1.60	(0.97, 2.61)	0.064
Female				1.00			1.00		
Age at initial diagnosis									
0–4 years	1.00			1.00			1.00		
5–9 years	1.53	(0.72, 3.24)	0.268	1.06	(0.27, 4.15)	0.938	1.37	(0.71, 2.64)	0.354
10–14 years	1.37	(0.59, 3.19)	0.472	3.10	(1.02, 9.37)	0.045	1.85	(0.96, 3.58)	0.066
15+ years	1.25	(0.45, 3.43)	0.671	4.44	(1.45, 13.59)	0.009	2.21	(1.08, 4.51)	0.030
Anthracycline Cumulative Dose (mg/m^2^)									
None	1.00			1.00			1.00		
1–100	1.13	(0.43, 2.95)	0.811	1.86	(0.43, 8.08)	0.409	1.43	(0.64, 3.20)	0.380
101–300	0.59	(0.23, 1.53)	0.278	2.74	(0.91, 8.29)	0.074	1.08	(0.52, 2.24)	0.829
>300	0.68	(0.21, 2.18)	0.516	2.17	(0.62, 7.63)	0.227	1.09	(0.46, 2.58)	0.839
Cyclophosphamide Equivalent Dose (mg/m^2^)									
None	1.00			1.00			1.00		
1–3999	0.85	(0.26, 2.80)	0.794	0.78	(0.21, 2.93)	0.711	0.78	(0.32, 1.92)	0.595
4000–7999	1.42	(0.51, 4.01)	0.504	0.41	(0.09, 1.79)	0.234	0.88	(0.38, 2.04)	0.768
>8000	1.76	(0.77, 4.00)	0.179	0.68	(0.25, 1.83)	0.444	1.24	(0.67, 2.30)	0.498
Epipodophyllotoxin Cumulative Dose (mg/m^2^)									
None	1.00			1.00			1.00		
1–1000	1.17	(0.31, 4.45)	0.817	0.62	(0.03, 11.60)	0.749	0.86	(0.25, 2.99)	0.810
1001–4000	1.45	(0.50, 4.17)	0.493	1.07	(0.18, 6.41)	0.944	1.22	(0.49, 3.00)	0.669
>4000	4.73	(1.76, 12.72)	0.002	5.08	(1.27, 20.37)	0.022	4.51	(2.04, 9.94)	<0.001
Platinum Cumulative Dose (mg/m^2^)									
None	1.00						1.00		
1–400	0.86	(0.19, 3.85)	0.847				0.79	(0.19, 3.40)	0.754
401–750	0.46	(0.06, 3.54)	0.453				0.40	(0.05, 2.98)	0.371
>750	1.42	(0.39, 5.23)	0.598				1.56	(0.45, 5.39)	0.484
Splenectomy									
Yes	2.56	(0.82, 7.98)	0.105	1.79	(0.54, 5.91)	0.339	2.03	(0.89, 4.62)	0.091
No	1.00			1.00			1.00		
Hematopoietic cell transplantation									
Yes	8.48	(3.98, 18.05)	<0.001				5.37	(2.72, 10.62)	<0.001
No	1.00						1.00		

The majority of survivors who developed late or very late subsequent leukemia survived less than five years from subsequent leukemia diagnosis with median survival of 0.78 years (range 0.03–26.44 years) and 2.88 years (range 0–17.31 years), respectively. No difference in duration of survival after subsequent leukemia diagnosis was observed between the two groups (*p =* 0.612). Subsequent leukemia was the most frequent etiology of death for survivors following LL and VLL (Table 2). Relative risk for all‐cause mortality was elevated following LL (RR 5.5, 95% CI 3.67–6.72) and VLL (RR 5.70, 95% CI 3.10–8.66) compared to survivors who did not develop subsequent leukemia.

## 4 DISCUSSION

We investigated the risk of subsequent LL and VLL among survivors of childhood cancer and report, to our understanding, the largest numbers of subsequent leukemias with associated treatment exposure data to date. Other studies of survivors of pediatric cancers have described subsequent late and very late leukemias^15^, ^17^; however, treatment‐associated risk factors could not be investigated due to limitations in number of cases^15^ and the availability of comprehensive treatment data.^17^ Within the CCSS cohort, survivors had a nine‐fold greater risk of developing subsequent late leukemia compared to the general population, an increase from previous reports^3^, ^15^, ^17^, ^28^ and the persistent risk for VLL is in contrast to other reports that have suggested subsequent leukemia risk plateaus after 10 years from initial diagnosis.^29^, ^30^ Survivors had increased risk for developing AML and MDS, similar to reports from prior studies,^15^, ^17^ as well as CML, which had been noted in one prior study, as well.^17^ The PanCareSurFup analysis included a larger number of survivors (*N* = 69,460) treated over a longer period of time (1940–2008) with a larger number of reported subsequent leukemias (*n* = 115), and similar to our study the majority were myeloid.^17^ Although that study identified similar primary diagnoses associated with risk, the magnitude of risk was smaller compared with the present study.^17^ It may be that among survivors treated in earlier treatment eras with the PanCareSurFup population, the intensity of chemotherapy treatment was less or the types of treatments were different. Similar to our analysis, the PanCare group reported that subsequent leukemias were occurring beyond 20 years from childhood cancer diagnosis.^17^ To our knowledge, our study is the first to demonstrate a persistent risk of LL and VLL among survivors who received treatment with high cumulative doses of epipodophyllotoxins.

There is extensive literature describing short latency subsequent leukemia associated with treatment exposures, and current survivorship guidelines from the Children's Oncology Group recommend monitoring for subsequent leukemia annually up to 10 years after exposure to epipodophyllotoxins and alkylating agents.^3^, ^29^, ^30^, ^31^ However, our study demonstrates a plateau does not exist and continued surveillance may be warranted beyond 10 years, particularly for high‐risk populations. In particular, our study may have important implications for surveillance screening in long‐term survivors where cumulative epipodophyllotoxin exposure is ≥ 4 gm/m^2^, which demonstrated in our study to result in a persistent risk for LL and VLL, and may be an important consideration for future treatment protocols and follow‐up guidelines.

The etiology for this longer latency is not fully elucidated and may be multi‐factorial. One of the strengths of the CCSS cohort is the duration of long‐term follow‐up, which may explain why our study did not demonstrate a plateau in secondary leukemia risk. Treatment exposures may cause alterations in oncogenes or tumor suppressor genes resulting in a “first hit,” and a secondary exposure is necessary for a “second hit” prior to development of subsequent leukemia. As an example, shortened telomere length was observed in survivors of lymphoma who underwent autologous HCT and developed subsequent MDS/AML, thus resulting in a “first hit.”^32^ As lifestyle factors change and treatment strategies are further refined, additional studies may determine the significance of this latency period and determine if this two‐hit hypothesis, similar to that of de novo leukemia, holds true in secondary leukemias. Additionally, survivors may have underlying cancer predisposition syndromes, resulting in secondary leukemia; specifically, survivors with an initial diagnosis of osteosarcoma followed by subsequent leukemia may be associated with an underlying p53 mutation. Furthermore, given that the majority of the survivors who developed a secondary leukemia had a primary hematologic malignancy, it is possible that there may be an underlying genetic propensity to developing hematologic malignancies, which is not yet established. Among the 77 survivors in our cohort who developed late subsequent leukemia, only three had an underlying genetic syndrome (Trisomy 21, nevoid basal cell carcinoma syndrome, and one “other” genetic disorder). As genetic evaluation becomes more accessible, subpopulations at highest risk may become evident and will allow refinement of surveillance recommendations.

Similar to prior studies,^17^ an association was observed between primary hematologic malignancy diagnoses and increased risk for subsequent leukemia. This could be a result of the fact that survival of hematologic malignancies improved earlier than other childhood malignancies, leaving more survivors reaching higher attained ages. It is also feasible immune dysregulation plays a part in development of subsequent leukemia, as there are known associations for the development of lymphoma (Hodgkin and NonHodgkin) in young children and underlying immune dysregulation.^33^ ^−^ ^34^ Though an association with splenectomy and development of subsequent leukemia was not observed in this study, it is possible that this was due to the limited number of cases that underwent splenectomy during treatment, as the overall trend for an association was seen, though significance was not established. This supports the possibility of immune dysregulation playing a role in the development of subsequent leukemia. Nevertheless, it is notable that treatment for hematologic malignancies in previous treatment eras was significantly different from contemporary protocols. Risk for LL and VLL reported here may not be observed among survivors of hematologic malignancies treated with more contemporary treatment protocols,^35^ particularly as lower cumulative doses of epipodophyllotoxins and alkylating agents are used. However, there is an increasing use of HCT in contemporary treatment. It is therefore feasible that survivors of hematologic malignancies may continue to be at risk for LL and VLL, as our study demonstrated an association between LL and HCT.

Interestingly, an increased risk for subsequent leukemia after Ewing sarcoma was not observed, which in contemporary treatment protocols includes exposure to high cumulative doses of epipodophyllotoxins and alkylating agents. There have been multiple studies describing short latency treatment‐related leukemia after treatment for Ewing sarcoma.^31^, ^36^, ^37^ The high rate of short latency treatment‐related leukemia after treatment for Ewing sarcoma, may contribute to lower numbers of late leukemias. Additionally, as Ewing sarcoma treatment has intensified in more contemporary protocols, CCSS survivors may experience different risk.

One goal of this study was to assess the association between smoking exposure and subsequent leukemia. Despite previously described associations between smoking and de novo AML in adults,^38^ we could not assess for an association between smoking and subsequent leukemia. This lack of association in our study may be due to how smoke exposure is measured in the CCSS cohort, as well as under self‐reporting and/or misclassification. The measurement does not account for dose–response or pack‐years, which could have resulted in different findings. Additionally, in the CCSS cohort smoking prevalence is lower than that seen in previous studies, which could reflect changing societal norms, limiting the power of our study to discern an association.

Limitations of this study must also be mentioned. Although the CCSS is a sizeable and well‐described cohort, it clearly does not include all childhood cancer survivors and there is a potential for participation bias. Subsequent neoplasms are initially self‐reported, which may lead to missed identification of impacted survivors. Additionally, the outcome of interest occurs many years after initial diagnosis, so loss to follow‐up may result in bias and underreporting. Furthermore, 7 of the subsequent leukemia (3 LL, 4 VLL) cases were preceded by a different SMN after childhood cancer diagnosis, and thus exposures from other SMN treatments or possibly predisposition for cancer development may have contributed to the development of subsequent leukemia in these cases, which was outside the scope of this current study. Moreover, cytogenetic data was not available for nearly 50% of cases and thus a more detailed classification of these secondary leukemias was not feasible. Finally, the CCSS cohort consists of patients treated between 1970 and 1999, and as such conclusions from this study may not apply to survivors who were treated on more contemporary treatment protocols or with more contemporary treatment modalities (i.e., immunotherapy, targeted agents).

In summary, survivors of childhood cancer are at increased risk of subsequent late and very late leukemia compared to the general population, specifically following exposure to high dose epipodophyllotoxins. Risk for subsequent leukemia does not plateau as previously suggested, and continued surveillance may be warranted in select populations, particularly those who have received ≥ 4 g/m^2^ cumulative dose of epipodophyllotoxins. An association between smoking history and subsequent late or very late leukemia was not observed, although other lifestyle factors such as obesity, which is a known risk factor in the general population for hematologic malignancies, including AML and ALL, may warrant study.^39^, ^40^, ^41^ Additionally, as our understanding of underlying genetic and lifestyle risks evolve, it may be beneficial to develop tailored surveillance strategies for survivors at highest risk for subsequent leukemia, including utilizing methods such as circulating tumor DNA (ctDNA). Though overall cumulative incidence for subsequent leukemia is not high, it continues to increase beyond 20 years from initial diagnosis and carries a high mortality burden, supporting the need for monitoring, educating, and counseling high‐risk survivors and their caregivers.

## AUTHOR CONTRIBUTIONS

**Taumoha Ghosh:** Conceptualization (equal); investigation (lead); writing – original draft (lead); writing – review and editing (lead). **Geehong Hyun:** Data curation (equal); formal analysis (equal); investigation (equal); methodology (equal); writing – review and editing (equal). **Rikeenkumar Dhaduk:** Data curation (equal); writing – review and editing (equal). **Miriam Conces:** Writing – review and editing (supporting). **Michael A. Arnold:** Writing – review and editing (supporting). **Rebecca M. Howell:** Methodology (supporting); writing – review and editing (supporting). **Tara O. Henderson:** Writing – review and editing (supporting). **Aaron McDonald:** Writing – review and editing (supporting). **Leslie L. Robison:** Conceptualization (supporting); writing – review and editing (supporting). **Yutaka Yasui:** Data curation (supporting); methodology (supporting); writing – review and editing (supporting). **Kirsten K. Ness:** Data curation (supporting); formal analysis (supporting); methodology (equal); supervision (supporting); writing – review and editing (supporting). **Gregory T. Armstrong:** Conceptualization (supporting); funding acquisition (lead); project administration (supporting); resources (equal); writing – review and editing (supporting). **Joseph P. Neglia:** Writing – review and editing (supporting). **Lucie M. Turcotte:** Conceptualization (equal); data curation (supporting); formal analysis (supporting); investigation (equal); methodology (supporting); supervision (lead); writing – review and editing (equal).

## FUNDING INFORMATION

This work was supported by the National Cancer Institute (CA55727, G.T. Armstrong, Principal Investigator). Support to St. Jude Children's Research Hospital also provided by the Cancer Center Support (CORE) grant (CA21765, C. Roberts, Principal Investigator) and the American Lebanese‐Syrian Associated Charities (ALSAC). The *funders* had no *role* in study design, data collection and analysis, decision to publish, or preparation of the *manuscript*.

## CONFLICT OF INTEREST STATEMENT

All other authors of this manuscript certify that they have NO affiliations with or involvement in any organization or entity with any financial interest (such as honoraria; educational grants; participation in speakers' bureaus; membership, employment, consultancies, stock ownership, or other equity interest; and expert testimony or patent‐licensing arrangements), or nonfinancial interest (such as personal or professional relationships, affiliations, knowledge or beliefs) in the subject matter or materials discussed in this manuscript.

## IRB STATEMENT

Approval from the human subjects committee was granted at participating institutions before participants were recruited, and participants provided informed consent.

## Data Availability

The Childhood Cancer Survivor Study is a US National Cancer Institute funded resource (U24 CA55727) to promote and facilitate research among long‐term survivors of cancer diagnosed during childhood and adolescence. CCSS data are publicly available on dbGaP at https://www.ncbi.nlm.nih.gov/gap/ through its accession number phs001327.v2.p1. and on the St Jude Survivorship Portal within the St. Jude Cloud at https://survivorship.stjude.cloud/. In addition, utilization of the CCSS data that leverages the expertise of CCSS Statistical and Survivorship research and resources will be considered on a case‐by case basis. For this utilization, a research Application Of Intent followed by an Analysis Concept Proposal must be submitted for evaluation by the CCSS Publications Committee. Users interested in utilizing this resource are encouraged to visit http://ccss.stjude.org. Full analytical data sets associated with CCSS publications since January of 2023 are also available on the St. Jude Survivorship Portal at https://viz.stjude.cloud/community/cancer‐survivorship‐community~4/publications.
